# Risk of childhood neoplasms related to neonatal phototherapy- a systematic review and meta-analysis

**DOI:** 10.1038/s41390-024-03191-7

**Published:** 2024-04-13

**Authors:** Ilari Kuitunen, Atte Nikkilä, Panu Kiviranta, Johanna Jääskeläinen, Anssi Auvinen

**Affiliations:** 1 University of Eastern Finland, Institute of Clinical Medicine and Department of Pediatrics, Kuopio, Finland; 2 Kuopio University Hospital, Department of Pediatrics, Kuopio, Finland; 3 Tampere University, Faculty of Medicine and Health Technologies, Tampere, Finland; 4 Kanta-Häme Central Hospital, Department of Pediatrics, Hämeenlinna, Finland; 5 The Finnish Medical Society Duodecim, Helsinki, Finland; 6 Tampere University, Faculty of Social Sciences, Department of Epidemiology, Tampere, Finland

## Abstract

**Context:**

Observational studies have shown conflicting results as to whether exposure to neonatal phototherapy is associated with increased rates of childhood cancer.

**Objective:**

To describe the rates of childhood neoplasms and cancer after neonatal phototherapy.

**Data sources:**

The CENTRAL, PubMed, Scopus, and Web of Science databases.

**Study selection:**

Observational studies regardless of design were included.

**Data extraction:**

The data were extracted by one author and validated by another. The risk-of-bias assessment was performed using the ROBINS-E and Joanna Briggs Institute critical appraisal tools.

**Results:**

Six cohort and 10 case-control studies were included. The overall risk of bias was high in seven and low in nine studies. In cohort studies, the odds ratio (OR) was increased for hematopoietic cancer (1.44; confidence interval [CI]: 1.16–1.80) and solid tumors (OR: 1.18; CI: 1.00–1.40). In case-control studies, the OR was 1.63 (CI: 0.99–2.67) for hematopoietic cancers and 1.18 (CI: 1.04–1.34) for solid tumors.

**Conclusions:**

Children with a history of neonatal phototherapy had increased risk of hematopoietic cancer and solid tumors. The evidence quality was limited due to the high risk of bias and potential residual confounding.

**Impact statement:**

- Exposure to neonatal phototherapy increased later risk of hematopoietic cancer and solid tumors.
- This is the most comprehensive study on the association between phototherapy and cancer, but the evidence quality was limited due risk of bias and residual confounding.
- Future large scale well conducted studies are still needed to better estimate the association and.

## Introduction

Neonatal jaundice is a common condition during the first month of life, as approximately 70% of neonates have some level of jaundice, and 5% to 10% require phototherapy for treatment of unconjugated hyperbilirubinemia.^1–3^ Phototherapy is commonly used to decrease bilirubin levels in order to avoid the neurotoxic effects of high bilirubin levels. Some of the known risk factors for unconjugated hyperbilirubinemia requiring phototherapy are maternal red blood cell antibodies, prematurity, birth injuries, hereditary factors (ethnicity and a history of phototherapy in older siblings), and maternal obesity.^3–5^

Phototherapy has been associated with some short-term adverse events, such as rash, dehydration, and difficulties with breastfeeding,^6,7^ as well as with long-term risks, such as allergies and seizure disorders.^8–10^ Phototherapy has been suggested to cause DNA damage and promote reactive oxygen species and proinflammatory cytokines, which could lead to an increased cancer risk.^11^ In addition, phototherapy has been associated with increased incidence of café-au-lait macules in children but not with melanocytic nevi.^12,13^ Previous studies have shown conflicting results regarding the possible increased incidence of childhood cancers following neonatal phototherapy. In some cohort studies, children exposed to phototherapy had an increased risk of all childhood cancers,^14,15^ whereas no such excess was reported in other studies.^16,17^ It has also been speculated that there may be an association between hyperbilirubinemia and malignancies. Therefore, the association between phototherapy may be due to higher bilirubin levels or other maternal/neonatal factors that increase the risk for both hyperbilirubinemia and neoplasms. As phototherapy is an effective and frequently used therapy for neonatal unconjugated hyperbilirubinemia,^18^ evidence summaries on possible long-term risk are of clinical relevance. A recent meta-analysis reported an increased risk for solid cancers among children treated with phototherapy, but the authors included benign nevi in their analysis and pooled case-control and cohort studies together, which caused a notable heterogeneity in their results.^19^ The aim of this systematic review was to provide a systematic assessment of the incidence of cancer and neoplasms after neonatal phototherapy.

## Methods

### Search process

The literature search was performed on June 28, 2022. We searched the PubMed (MEDLINE), Web of Science, CENTRAL, and Scopus databases for these search terms: (neonat* OR newborn* OR infant*) AND (phototherapy OR hyperbilirubinemia OR jaundice) AND (cancer or malign* OR leukemia OR leukaemia OR lymphoma* OR tumor* or neoplasm*). Additional articles were included if found in the references of the included articles and assessed suitable for the review and analysis. We did not search other sources and decided not to include gray literature. The full search strategy is presented in the appendix (Supplementary file 1).

### Inclusion criteria

We included only human studies published in peer-reviewed journals in English. Retrospective and prospective observational studies with control groups, regardless of the design (cohort, case-control, etc.) were included. Studies focusing on benign and/or malignant neoplasms, leukemia, and lymphomas were included.

### Exclusion criteria

We excluded studies focusing only on nevi or other benign tumors (including hemangiomas). All animal studies were also excluded. Studies without original data or reported in languages other than English were excluded as well.

### Main outcome

Our main outcome was neoplasm and cancer risk estimates stratified by anatomic site and the cell type of the neoplasm. We aimed to collect the mortality due to cancers.

### Data extraction

Two authors screened the abstracts and full texts using Covidence software.^20^ A third party was consulted in cases of disagreement if mutual consensus was not achieved. Data extraction was performed by one author and validated by another. The following information was extracted to a pre-designed spreadsheet: authors, year of publication, country where the study was conducted, study period, study design, original inclusion criteria, exposure and control, total number of people included in the study, number of exposed and unexposed or number of cases and controls (depending on the study design), follow-up duration, and overall person-years of follow-up. The effect estimates from both adjusted and unadjusted analyses (hazard ratios [HRs], incidence rates, odds ratio [ORs], and risk ratios [RRs]) with uncertainty estimates (95% confidence intervals [CIs]) were abstracted as well.

### Risk-of-bias assessment

Risk of bias was assessed for all the included studies. We used the Risk of Bias in Non-randomized Studies of Exposures (ROBINS-E) tool to assess risk of bias.^21^ If the study did not attempt to adjust for confounding, it was immediately labeled as high risk for bias, and other domains were not assessed. The scale used in the judgment was *low*, *some concerns*, and *high*. We also utilized a secondary risk-of-bias assessment strategy. We analyzed the cohort studies’ risk of bias according to the Joanna Briggs Institute critical appraisal tool for cohort studies and the case-control studies’ according to the Joanna Briggs Institute critical appraisal tool for case-control studies.^22^ These were labeled as *with concerns* or *no concerns*. We decided not to exclude any reports from the synthesis due to risk of bias but performed sensitivity analyses where these were excluded.

### Statistical methods

RevMan version 5.4 and R statistical software version 4.2.2 (metafor package) were used for the meta-analysis. Data analysis was performed according to *Cochrane Handbook for Systematic Reviews* guidelines. Forest plots are presented for all outcomes.

We decided not to pool case-control studies with cohort studies, as these have different inclusion strategies and are thus problematic to combine. Overall, we expected heterogeneity in the populations between the studies, and therefore we decided to use the random-effects Mantel-Haenszel model.^23^ Pooled ORs with 95% CIs were calculated with the Mantel-Haenszel method for cohort and case-control studies. The inconsistency index statistic I² for statistical heterogeneity was calculated, but it was not used to decide whether the fixed-effect or random-effect model was used. Some of the studies contained outcomes that could not be pooled for quantitative analysis, and these outcomes have been reported according to the Synthesis Without Meta-analysis (SWiM) guideline.^24^ For example, the adjusted effect estimates in the included studies had high heterogeneity (confounder selection, statistical method, chosen effect estimate measure [OR, RR, HR]) in the reporting, and thus we decided not to force this to a single estimate and presented these in a table. We assessed publication bias by Egger’s test and the trim and fill method and provide the funnel plots.^25^

We report our meta-analysis according to the Meta-analysis of Observational Studies in Epidemiology (MOOSE) and Preferred Reporting Items in Systematic Reviews and Meta-analyses (PRISMA) guidelines and provide the checklists in the appendix.^26,27^

### Protocol registration

We registered our protocol in Prospero (ID CRD42022342273), and it can be assessed online: https://www.crd.york.ac.uk/prospero/display_record.php?ID=CRD42022342273.

## Results

**Fig. 1:**
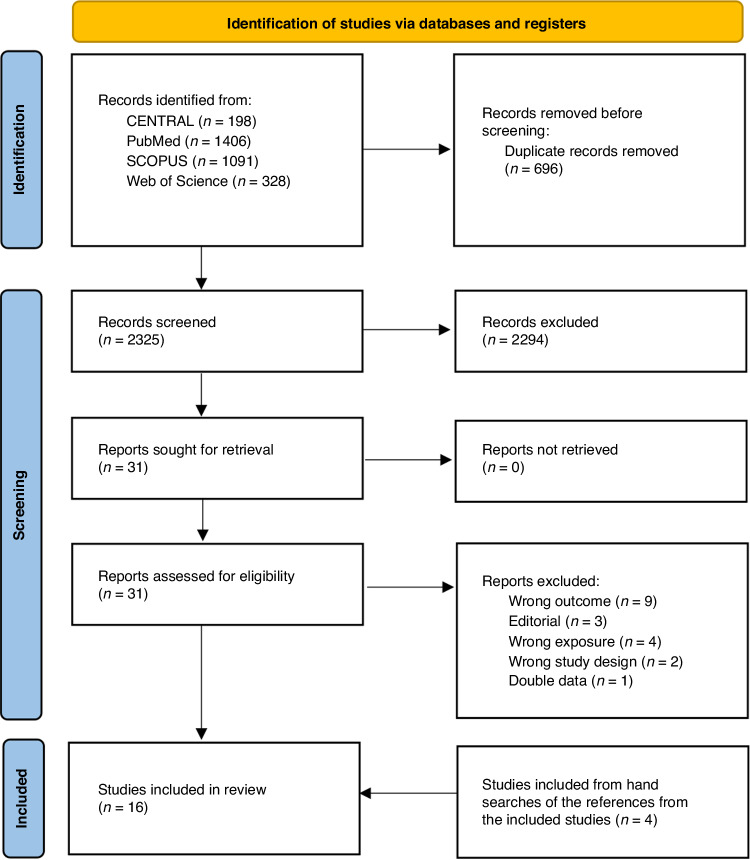
PRISMA flowchart of the study selection process.

We initially screened 2,325 abstracts and assessed 31 full reports. After exclusions (19 studies) and inclusions from hand searches (4 studies), a total 16 studies were included for systematic review and meta-analysis Figure 1.^14–16,28–40^ Six were retrospective cohort studies and 10 case-control studies (Table 1). Eight of the studies were from Europe, five from North America, and three from the Middle East. The study periods ranged from the 1960s to the 2010s. The main outcome used was the odds or risk of any cancer. The number of participants varied between 150 and 0.9 million (Table 2). Six studies did not adjust their analysis, and, furthermore, only five studies described a rationale for the selection of the covariates for adjustments (Table 2).

**Table 1:** Background characteristics of the included studies

Study	Study period	Country	Study design	Data coverage	Follow-up	Exposure classification	Main outcomes
Auger et al. ^15^	2006-2016	Canada	cohort	regional	retrospective	Binary	All cancer
Berg et al. ^29^	1973-1992	Sweden	case-control	nationwide	retrospective	Binary	Melanoma
Brewster et al. ^30^	1976-2006	Scotland	cohort	regional	retrospective	Binary	Skin cancer
Bugaiski-Shaked et al. ^31^	1988-2018	Israel	cohort	institutional	retrospective	Binary	All cancer
Cnattingius 1995a	1973-1989	Sweden	case-control	nationwide	retrospective	Binary	Myeloid leukemia
Cnattingius 1995b	1973-1989	Sweden	case-control	nationwide	retrospective	Binary	Lymphatic leukemia
Digitale et al. ^16^	1995-2017	USA	cohort	regional	retrospective	Binary	All cancer
Heck et al. ^33^	1977-2013	Denmark	case-control	nationwide	retrospective	Binary	All cancer
Kadivar et al. ^34^	2015-2018	Iran	case-control	institutional	retrospective	Binary and duration	All cancer
Linet et al. ^38^	1973-1989	Sweden	case-control	nationwide	retrospective	Binary	Brain tumors
Olsen et al. ^44^	1977-1989	Denmark	cohort	national	retrospective	Binary	All cancer
Podvin et al. ^40^	1981-2003	USA	case-control	regional	retrospective	Binary	Leukemia
Roman et al. ^39^	1962-1996	England	case-control	regional	retrospective	Binary	Hematopoietic cancer
Sabzevari et al. ^35^	2011-2018	Iran	case-control	institutional	retrospective	Binary and duration	All cancer
Seppälä et al. ^36^	1996-2014	Finland	case-control	nationwide	retrospective	Binary	All cancer
Wickremasinghe et al. ^14^	1998-2007	USA	cohort	regional	retrospective	Binary	All cancer

**Table 2:** Study characteristics of the included studies.

STUDY	Follow-up length (years)	Overall N of participants	Adjustment variates	Variates chosen by
Auger et al. ^15^	Maximum 17 years	548	Maternal age, gestational age, birth weight, multiple birth, cesarean section, infant sex, socioeconomic deprivation, place of residence, and birth year	Not specified why.
Berg et al. ^29^	Not specified	12,138	No adjustments	Not applicable.
Brewster et al. ^30^	Not specified	201,977	No adjustments	Not applicable.
Bugaiski-Shaked et al. ^31^	median 18 years	150	Preterm birth, maternal age	Known risk factors were tried (potential confounders). *P* < 0.1 led to inclusion in the model. Subgroup analyses were carried out excluding some known risk factors of childhood cancer (multiple gestations, prematurity, malformations)
Cnattingius 1995a	Maximum 19 years	588	No adjustments	Not applicable
Cnattingus 1995b	Maximum 19 years	3,690	No adjustments	Not applicable
Digitale et al. ^16^	Maximum 11 years	786,998	sex, race, gestational age, delivery mode, facility of birth, year of birth, maternal age, multiple birth, birth weight, chromosomal abnormalities, early jaundice, bilirubin, Down syndrome, congenital abnormalities	Hypothesized confounders included. Down syndrome not in the propensity model of hematopoietic cancers.
Heck et al. ^33^	median 24 years	77,528	maternal age, birth weight, gestational age, sex, and birth year	Like to Auger et al. 2019, as the aim was to reproduce their analyses with Danish data.
Kadivar et al. ^34^	median 9.5 years	342,172	sex, father’s smoking, mother’s age during pregnancy	Unclear. It was mentioned that birth weight was excluded from the model due to collinearity. Data was available for many other variables.
Linet et al. ^38^	Maximum 19 years	3420	No adjustments	Not applicable
Olsen et al. ^44^	Not specified	232	Age, sex and time period	Based on availability, no specific explanation given.
Podvin et al. ^40^	Maximum 19 years	6545	child’s race/ethnicity, birthweight, sex, gestational age, maternal and paternal age, maternal smoking, maternal diabetes, maternal marital status and parity	Potential confounders with at least 10% effect to estimate.
Roman et al. ^39^	Not specified	429	No adjustments	Not applicable.
Sabzevari et al. ^35^	Cases mean 6.9 years Controls mean 4.4 years	1000	Maternal age, mother’s educational level, history of radiographs taken, history of maternal infection	Not specified why.
Seppälä et al. ^36^	Mean 9.1 years	55,120	maternal age, parity and smoking	Not specified why.
Wickremasinghe et al. ^14^	Mean 8.2 years	139,100	Propensity-adjusted model with following variables: sex, birth weight, gestational age, large for gestational age, twin birth, birth by cesarean delivery, payer source, year of birth, maternal race, paternal race, maternal age, paternal age, maternal education, paternal education, Down syndrome, other chromosomal and nonchromosomal anomalies.	Not specified why.

### Risk of bias and publication bias

Risk of bias was assessed by ROBINS-E; nine studies were judged to have a low risk of bias, and seven studies had a high risk of bias due to lack of adjustment for potential confounders (Table 3). Concerns were found in nine studies with the Joanna Briggs Institute critical appraisal tool. Most issues were in confounder identification and strategies to address incomplete follow-up in cohort studies. In case-control studies, most issues were in measuring the exposure and appropriate statistical analysis (Table 3). We did not detect publication bias visually in funnel plots, and Egger’s test confirmed this. The trim and fill method was utilized and showed no obvious asymmetry (Fig. S1).

**Table 3:** Risk of bias assessment according to Risk of bias in observational studies of exposures (ROBINS-E) and Joanna Briggs Institute Critical appraisal tools for cohort and case-control studies.

	ROBINS-E	Joanna Briggs Institute Critical Appraisal Tools											
	overall	Were the two groups similar and recruited from the same population?	Were the exposures measured similarly to assign people to both exposed and unexposed groups?	Was the exposure measured in a valid and reliable way?	Were confounding factors identified?	Were strategies to deal with confounding factors stated?	Were the groups/participants free of the outcome at the start of the study?	Were the outcomes measured in a valid and reliable way?	Was the follow up time reported and sufficient to be long enough for outcomes to occur?	Was follow up complete, and if not, were the reasons to loss to follow up described and explored?	Were strategies to address incomplete follow up utilized?	Were statistical analysis appropriate?	Overall
**COHORT STUDIES**													
Auger et al. ^15^	Low	Yes	Yes	Yes	Yes	Yes	Yes	Yes	Yes	Yes	Yes	Yes	Include
Brewster et al. ^30^	High*	No	Unclear	Yes	No	No	Yes	Yes	Yes	Yes	Unclear	No	Concerns
Bugaiski-Shaked et al. ^31^	Low	Yes	Yes	Yes	Yes	Yes	Yes	Yes	Yes	Yes	Yes	Yes	Include
Digitale et al. ^16^	Low	Yes	Yes	Yes	Yes	Yes	Yes	Yes	Yes	Yes	Yes	Yes	Include
Olsen 1996	High*	No	Unclear	Yes	No	No	Yes	Yes	Yes	Yes	Unclear	No	Concerns
Wickremasinghe et al. ^14^	Low	Yes	Yes	Yes	Yes	Yes	Yes	Yes	Yes	Yes	Yes	Yes	Include
**CASE-CONTROL STUDIES**			Were the groups comparable other than the presence of disease in cases or the absence of disease in controls?	Were cases and controls matched appropriately?	Were the same criteria used for identification of cases and controls?	Was exposure measured in a standard, valid and reliable way?	Was exposure measured in the same way for cases and controls?	Were confounding factors identified?	Were strategies to deal with confounding factors stated?	Were outcomes assessed in a standard, valid and reliable way for cases and controls?	Was the exposure period of interest long enough to be meaningful?	Were statistical analysis appropriate?	Overall
Berg et al. ^29^	High*		Yes	Yes	Yes	Unclear	Yes	No	No	Yes	Yes	Yes	Concerns
Cnattingius 1995a	High*		Yes	Yes	Yes	Unclear	Yes	Yes	Yes	Yes	Yes	Unclear	Concerns
Cnattingius 1995b	High*		Yes	Yes	Yes	Unclear	Yes	Yes	Yes.	Yes	Yes	Unclear	Concerns
Heck et al. ^33^	Low		Yes	Yes	Yes	Yes	Yes	Yes	Unclear	Yes	Yes	Yes	Include
Kadivar et al. ^34^	High*		No.	No.	No	Unclear	Yes	Unclear	No	Yes	Yes	No	Concerns
Linet et al. ^38^	High*		Yes	Yes	Yes	Unclear	Yes	Yes	Yes	Yes	Yes	Unclear	Concerns
Podvin et al. ^40^	Low		Yes	Yes	Yes	Yes	Yes	Yes	Yes	Yes	Yes	Yes	Include
Roman et al. ^39^	High*		Yes	Yes	Yes	Yes	Yes	No	Unclear	Yes	Yes	Unclear	Concerns
Sabzevari et al. ^35^	Low		Yes	Yes	No	Yes	Yes	Yes	No	Yes	Yes	No	Concerns
Seppälä et al. ^36^	Low		Yes	Yes	Yes	Yes	Yes	Yes	Yes	Yes	Yes	Yes	Include

^*^High due to lack of attempt to manage confounding.

### Cancer and tumor incidence in cohort studies

**Fig. 2:**
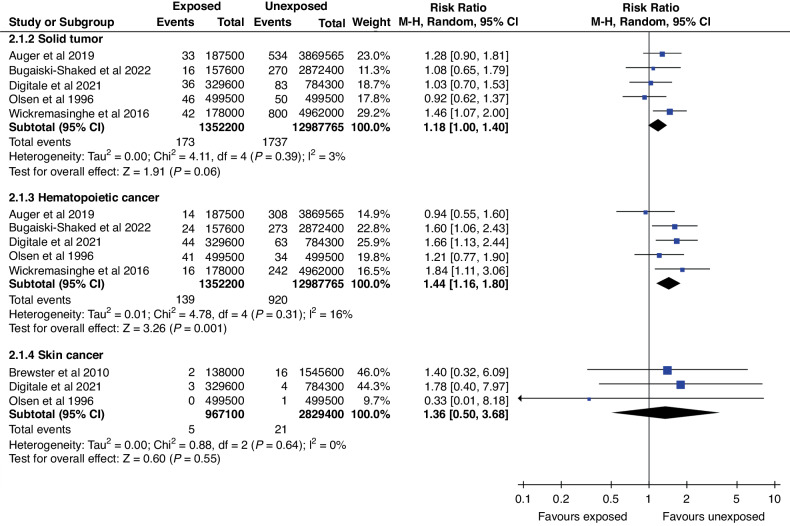
Forest plot of the cancer incidence between phototherapy exposed cohort and unexposed cohort stratified by the cancer type.

Six cohort studies with a combined follow-up of 16 million person-years were analyzed and pooled for all cancer incidence estimates. In analysis by cancer type, the risk of hematopoietic cancers (OR: 1.44; CI: 1.16–1.80) and solid tumors (OR: 1.18; CI: 1.00–1.40) was increased. Rates of solid tumors and skin cancers did not show evidence of difference in crude analysis (Fig. 2). In sensitivity analyses, in which studies with high risk of bias were excluded, the OR changed only for skin cancers, and risk remained highly imprecise (OR: 1.78; CI: 0.70–7.97) (Fig. S2).

In adjusted analyses of the cohort studies, statistically significant associations were detected in two studies regarding all cancer incidences (Table 4). In stratified analysis, one study found an increased overall adjusted hazard of hematopoietic cancers and one an increased adjusted OR (aOR) for acute myeloid leukemia. One study further presented an increased aOR for kidney cancer but not for any other type of solid cancer.

**Table 4:** Adjusted estimates for cancer from the original studies.

	All cancer	Hematopoietic cancer	Solid tumors	Skin cancer
**COHORT STUDIES**				
Auger et al. ^15^	aHR 1.34 (CI 0.99–1.83)	aHR 1.32 (CI 0.81–2.14)	aHR 1.36 (CI 0.91–2.02)	
Bugaiski-Shaked et al. ^31^	aHIR 1.89 (CI 1.35–2.67)	aHR 2.29 (CI 1.48–3.54)	aHR 1.37 (CI 0.82–2.29)	
Digitale et al. ^16^	aHR 1.13 (0.83–1.53)	aHR 1.17 (CI 0.74–1.83)	aHR 1.01 (CI 0.65–1.58)	aHR 4.13 (CI 0.88–19.43)
Wickremasinghe et al. ^14^	aOR 1.4 (CI 1.1–1.9)	ALL aOR 1.3 (0.6–2.9) AML aOR 2.6 (1.3–5.0)	Brain aOR 1.0 (CI 0.5–2.1) Kideny aOR 2.5 (CI 1.2–5.1) Liver aOR 0.6 (CI 0.2–2.5) Soft tissue aOR 0.4 (CI 0.1–2.7)	
**CASE-CONTROL STUDIES**				
Heck et al. ^33^		ALL aOR 1.69 (CI 1.37–2.08) AML aOR 1.45, CI (0.99–2.38) Lymphoma aOR 1.41 (0.95–2.10)		
Podvin et al. ^40^		aOR 1.4 (CI 0.5–3.9)		
Sabzevari et al. ^35^	aOR 1.38 (CI 0.03–55.53)			
Seppälä et al. ^36^	aOR 1.11 (CI 0.91–1.35)			

Most adjusted model selected from the each included study. Adjusted hazard ratios (aHR), adjusted odds ratios (aOR) with 95% confidence intervals (CI) presented.

### Cancer and tumors in case-control studies

**Fig. 3:**
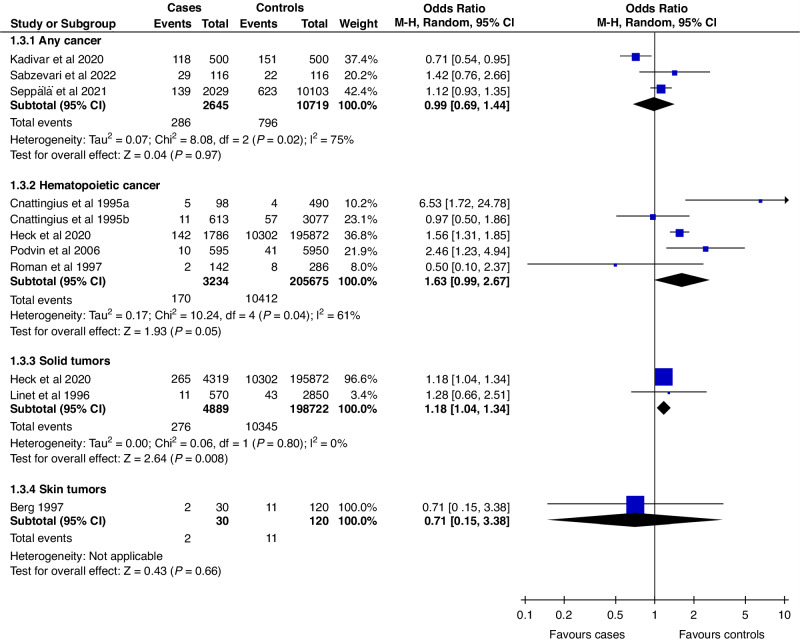
Forest plot of the crude overall cancer/ tumor rates of exposed and unexposed between case and control groups in case-control studies stratified by the tumor/cancer type.

Ten case-control studies were included for a pooled analysis with 10,799 cancer cases, of whom 734 (7.0%) had been exposed to phototherapy. The control group consisted of 219,364 children, of whom 11,262 (5.1%) were exposed to phototherapy. In the analysis by tumor type, solid tumors were the only group with increased risk associated with phototherapy (OR: 1.18; CI: 1.04–1.34) (Fig. 3). This estimate remained unchanged in sensitivity analysis (Fig. S3). The OR for hematopoietic cancers was 1.63 (CI: 0.99–2.67). In the sensitivity analysis, the OR for hematopoietic cancers was 1.70 (CI: 1.14–2.55) (Fig. S3), indicating increased odds, when only studies with a low risk of bias were included.

Four case-control studies presented adjusted analyses. In the adjusted analyses, the aOR was statistically significant in one study and for only one outcome. The acute lymphatic leukemia aOR was 1.69 (CI: 1.37–2.08). Other adjusted estimates had CIs overlapping 1 (Table 4).

## Discussion

### Main findings

Based on this systematic review and meta-analysis, children with a history of neonatal phototherapy have a 1.2- to 1.6-fold increased risk of hematopoietic cancers and solid tumors. However, several factors need to be considered in interpretation, including issues with the quality of reporting in the original studies, potential causal pathways, and confounding factors.

Some studies have speculated that the increased cancer risk could be at least partly attributable to hyperbilirubinemia instead of phototherapy, i.e., confounding by indication. This could be related to oxidative stress caused by bilirubin at the cellular level, which could promote carcinogenesis.^41^ This is consistent with findings showing that cancer incidence among children with hyperbilirubinemia who did not receive phototherapy was between that of children without hyperbilirubinemia and that of those treated with phototherapy.^15,40^

We originally intended to analyze cancer risk by duration and intensity of phototherapy, as it could be hypothesized that longer treatment duration could lead to higher risk. However, it turned out that most studies did not report the phototherapy duration.

Prematurity has been associated with both phototherapy and cancer risk. One of the included studies analyzed term and preterm infants separately and found that incidence did not differ between the treated and non-treated individuals who were born prematurely, whereas among full-term infants phototherapy was associated with a slightly increased risk of hematopoietic cancers.^33^

### Comparison to previous meta-analyses

During our initial search process, we identified a previous meta-analysis, and later another one was identified.^19,42^ Their results were generally similar to ours, but there were some key differences and issues in the previous meta-analyses. Both previous meta-analyses pooled case-control and cohort studies and reported their combined results. Although this is technically possible, it increases variability in study populations and adds to heterogeneity. Furthermore, the meta-analysis by Hemati et al. also included benign nevi count as an outcome and did not present any sensitivity analysis to assess the impact of risk of bias or reasons for high heterogeneity. Furthermore, we were able to include one additional study to the meta-analysis by Abdellatif et al.

### Strengths

We performed our systematic review according to a pre-registered protocol without major deviations. In contrast to previous studies, we did not pool results from case-control and cohort studies, which reduces the heterogeneity in our reporting. The results from case-control studies exhibited high variability, including both increased and decreased odds. Furthermore, the measured inconsistency was high. The effect estimates from cohort studies had lower heterogeneity, which was also seen as higher statistical consistency. It must be noted that, based on the wide CIs, nearly all the included studies seemed to be underpowered to detect meaningful risk increases.

### Limitations

Most of the limitations of this work come from the limitations of the included studies. Several studies had a high risk of bias due to lack of adjustment for possible confounders. The studies that did adjust for confounders rarely presented the rationale for the covariate selection. None of the studies discussed causal pathways or visualized them, e.g., as directed acyclic graphs. To overcome this issue, we have visualized the potential causal pathways in Figure S4 to better illustrate the possible causality and alternative backdoor paths causing bias to estimates.

We were unable to perform two analyses planned in the protocol: mortality and exposure-outcome gradient (dose dependency). As the studies did not report mortality, we were unable to assess it. Furthermore, we aimed to examine the exposure gradient (higher risk with higher exposure level) in the potential association, as it could have strengthened the plausibility of a potential effect. Dose dependency would have been addressed by examining the duration and intensity of the phototherapy, but only two studies presented information on duration and none on the intensity (number of lamps). Furthermore, we were unable to find information on the phototherapy practices in the included countries during the study periods, as there may have been variations in the bilirubin levels for phototherapy initiation and ending. Thus, this causes additional heterogeneity in our estimates.

### Implications for clinical practice and future research

Future studies are still needed. Although our systematic review identified 16 studies, the overall quality had clear limitations. Furthermore, due to the rare outcome, estimates in our meta-analysis have notable imprecision, and further large-scale studies are needed. Future studies should focus more on potential causal pathways in selecting the covariates for their analyses. We have illustrated the potential causal pathways and modifiers, which could partly explain the observed differences (Fig. S4). Some maternal and neonatal conditions, such as prematurity, congenital anomalies, hereditary syndromes, and intrauterine growth restrictions, may increase the rates of phototherapy and cancers. Inability to control for these creates a potential source of bias due to confounding by indication and shared risk factors. Mortality in cancer patients with and without prior phototherapy would be an interesting topic to address in the future.

While our results suggest that neonatal phototherapy may increase the risk of hematopoietic cancers and solid tumors, they do not justify changes in the use of phototherapy. As high bilirubin levels are neurotoxic, it is important to treat hyperbilirubinemia appropriately. However, guidelines should be followed and unnecessary therapy avoided, as it may have harmful effects.^43^ Currently, we cannot conclude whether the phototherapy, high bilirubin, or shared risk factors for prematurity and childhood cancer underlie the observed association with cancer risk.

## Conclusion

Neonates receiving phototherapy have a 1.2- to 1.6-fold increased risk of hematopoietic cancers and solid tumors. Quality concerns in the reporting of the original studies limited the evidence. More high-quality studies are needed to further elucidate the observed association between phototherapy and neoplasia and improve understanding of the potential causal pathways.

## Supplementary information

MOOSE-checklist

PRISMA_2020_checklist

Supplementary material

## Data Availability

All the data generated during the review process are available from the corresponding author upon request.
