# Supplementary material for: Risk of childhood neoplasms related to neonatal phototherapy- a systematic review and meta-analysis
Source: Pediatr Res. 2024 Apr 13;96(5):1131–40. doi: 10.1038/s41390-024-03191-7 (PMC11521995; doi:10.1038/s41390-024-03191-7)

Supplementary materials

Supplementary file 1: Search strategy

PubMed: ("neonat*"[All Fields] OR "newborn*"[All Fields] OR "infant*"[All Fields]) AND ("phototherapy"[MeSH Terms] OR "phototherapy"[All Fields] OR "phototherapies"[All Fields] OR ("hyperbilirubinaemia"[All Fields] OR "hyperbilirubinemia"[MeSH Terms] OR "hyperbilirubinemia"[All Fields] OR "hyperbilirubinaemias"[All Fields] OR "hyperbilirubinemias"[All Fields]) OR ("jaundice"[MeSH Terms] OR "jaundice"[All Fields] OR "jaundiced"[All Fields] OR "jaundices"[All Fields])) AND ("cancer s"[All Fields] OR "cancerated"[All Fields] OR "canceration"[All Fields] OR "cancerization"[All Fields] OR "cancerized"[All Fields] OR "cancerous"[All Fields] OR "neoplasms"[MeSH Terms] OR "neoplasms"[All Fields] OR "cancer"[All Fields] OR "cancers"[All Fields] OR "malign*"[All Fields] OR ("leukaemia"[All Fields] OR "leukemia"[MeSH Terms] OR "leukemia"[All Fields] OR "leukaemias"[All Fields] OR "leukemias"[All Fields] OR "leukemia s"[All Fields]) OR ("leukaemia"[All Fields] OR "leukemia"[MeSH Terms] OR "leukemia"[All Fields] OR "leukaemias"[All Fields] OR "leukemias"[All Fields] OR "leukemia s"[All Fields]) OR "lymphoma*"[All Fields] OR "tumor*"[All Fields] OR "neoplasm*"[All Fields])

SCOPUS: (neonat* or newborn* or infant*) AND (phototherapy or hyperbilirubinemia or jaundice) AND (cancer or malign* or leukemia or leukaemia or lymphoma* or tumor* or neoplasm*

CENTRAL: photoherapy and (cancer or tumor or neoplasm)

Web of Science: (phototherapy or hyperbilirubinemia or jaundice) and (cancer or tumor or neoplasm or leukemia or lymphoma) and (newborn or neonate or neonatal or infant or infants)

Publication bias

Supplementary figure 1 Funnel plots of included cohort studies (A) and case-control studies (B). Egger's test used for statistical testing. White area represents p>0.05. Trim and fill method was tried but it produced no new points to the figure as the included studies form an expectedly symmetric distribution

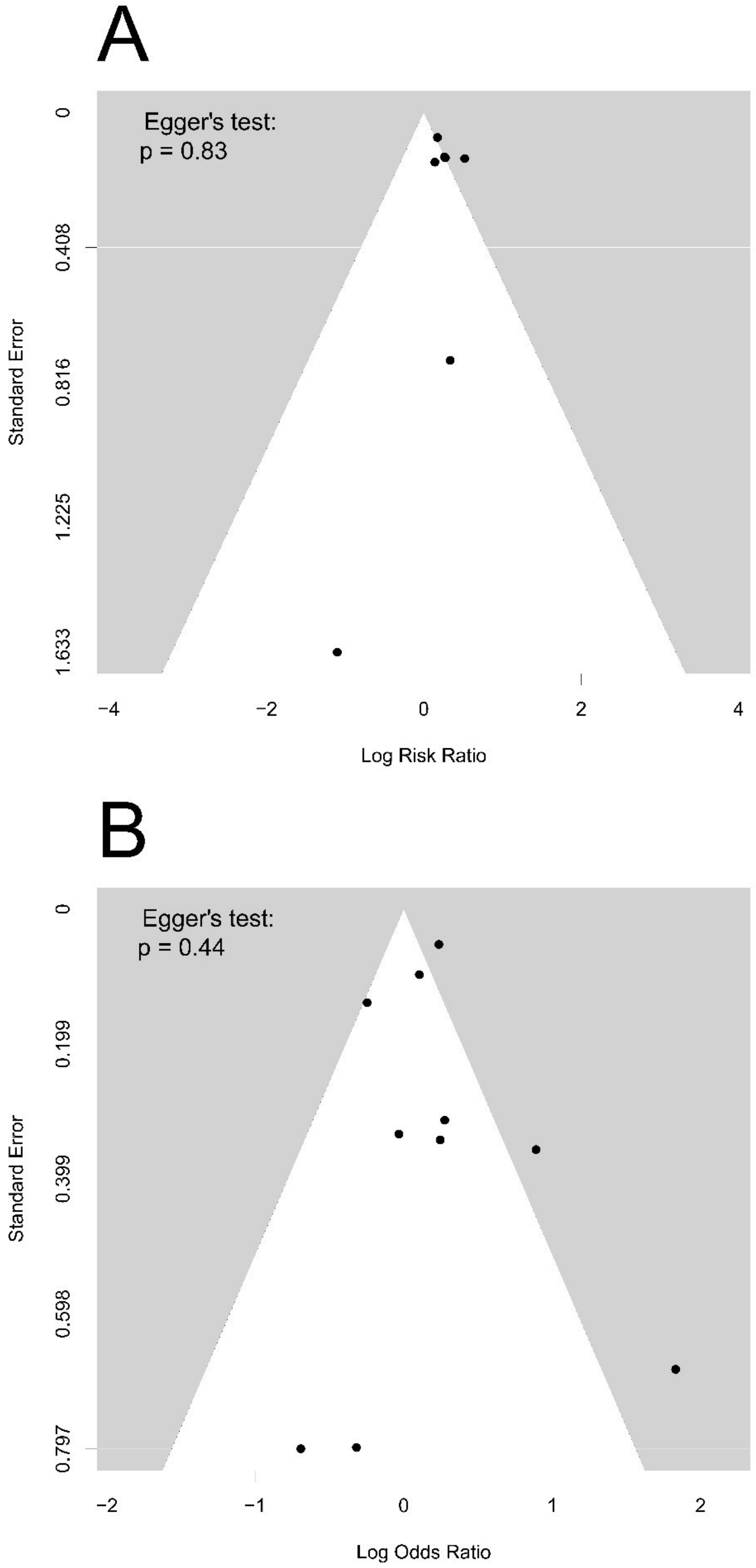

Sensitivity analysis for all main outcomes. Studies judged to be in high risk of bias were excluded.

Figure S2: Sensitivity analysis for the figure 2

Study or Subgroup	Exposed Events	Exposed Total	Unexposed Events	Unexposed Total	Weight	Risk Ratio M-H, Random, 95% CI
**2.1.2 Solid tumor**						
Auger et al 2019	33	187500	534	3869565	27.9%	1.28 [0.90, 1.81]
Bugaiski-Shaked et al 2022	16	157600	270	2872400	13.6%	1.08 [0.65, 1.79]
Digitale et al 2021	36	329600	83	784300	22.6%	1.03 [0.70, 1.53]
Olsen et al 1996	46	499500	50	499500	0.0%	0.92 [0.62, 1.37]
Wickremasinghe et al 2016	42	178000	800	4962000	35.9%	1.46 [1.07, 2.00]
**Subtotal (95% CI)**		**852700**		**12488265**	**100.0%**	**1.25 [1.04, 1.50]**
Total events	127		1687			
Heterogeneity: Tau² = 0.00; Chi² = 2.26, df = 3 (P = 0.52); I² = 0%						
Test for overall effect: Z = 2.34 (P = 0.02)						
**2.1.3 Hematopoietic cancer**						
Auger et al 2019	14	187500	308	3869565	19.2%	0.94 [0.55, 1.60]
Bugaiski-Shaked et al 2022	24	157600	273	2872400	28.2%	1.60 [1.06, 2.43]
Digitale et al 2021	44	329600	63	784300	31.6%	1.66 [1.13, 2.44]
Olsen et al 1996	41	499500	34	499500	0.0%	1.21 [0.77, 1.90]
Wickremasinghe et al 2016	16	178000	242	4962000	21.0%	1.84 [1.11, 3.06]
**Subtotal (95% CI)**		**852700**		**12488265**	**100.0%**	**1.51 [1.16, 1.95]**
Total events	98		886			
Heterogeneity: Tau² = 0.02; Chi² = 3.97, df = 3 (P = 0.26); I² = 25%						
Test for overall effect: Z = 3.08 (P = 0.002)						
**2.1.4 Skin cancer**						
Brewster et al 2010	2	138000	16	1545600	0.0%	1.40 [0.32, 6.09]
Digitale et al 2021	3	329600	4	784300	100.0%	1.78 [0.40, 7.97]
Olsen et al 1996	0	499500	1	499500	0.0%	0.33 [0.01, 8.18]
**Subtotal (95% CI)**		**329600**		**784300**	**100.0%**	**1.78 [0.40, 7.97]**
Total events	3		4			
Heterogeneity: Not applicable						
Test for overall effect: Z = 0.76 (P = 0.45)						

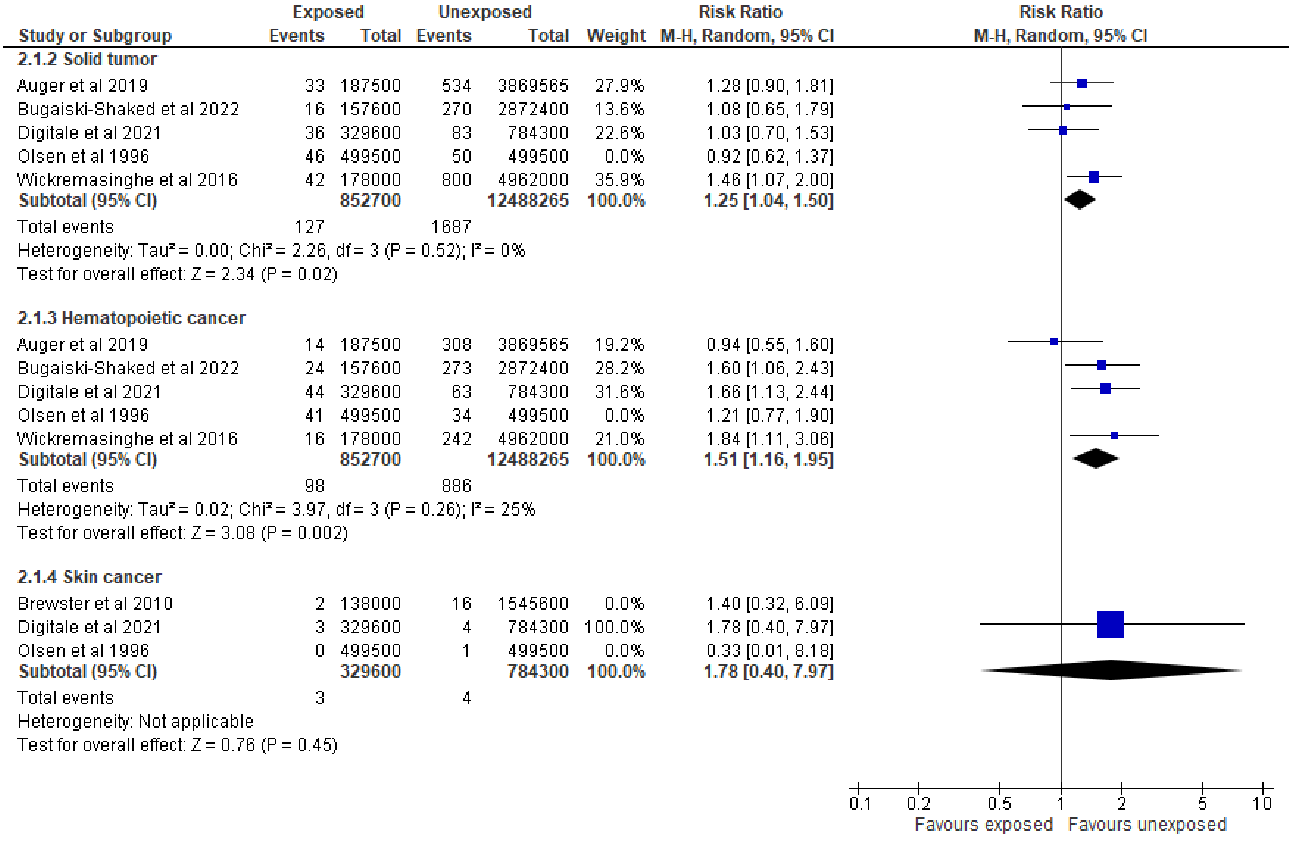

Figure S3: Sensitivity analysis of the figure 3

Study or Subgroup	Cases Events	Cases Total	Controls Events	Controls Total	Weight	Odds Ratio M-H, Random, 95% CI
**1.3.1 Any cancer**						
Kadivar et al 2020	118	500	151	500	0.0%	0.71 [0.54, 0.95]
Sabzevari et al 2022	29	116	22	116	8.5%	1.42 [0.76, 2.66]
Seppälä et al 2021	139	2029	623	10103	91.5%	1.12 [0.93, 1.35]
**Subtotal (95% CI)**		**2145**		**10219**	**100.0%**	**1.14 [0.95, 1.37]**
Total events	168		645			
Heterogeneity: Tau² = 0.00; Chi² = 0.52, df = 1 (P = 0.47); I² = 0%						
Test for overall effect: Z = 1.43 (P = 0.15)						
**1.3.2 Hematopoietic cancer**						
Cnattingius et al 1995a	5	98	4	490	0.0%	6.53 [1.72, 24.78]
Cnattingius et al 1995b	11	613	57	3077	0.0%	0.97 [0.50, 1.86]
Heck et al 2020	142	1786	10302	195872	78.0%	1.56 [1.31, 1.85]
Podvin et al 2006	10	595	41	5950	22.0%	2.46 [1.23, 4.94]
Roman et al 1997	2	142	8	286	0.0%	0.50 [0.10, 2.37]
**Subtotal (95% CI)**		**2381**		**201822**	**100.0%**	**1.72 [1.19, 2.50]**
Total events	152		10343			
Heterogeneity: Tau² = 0.04; Chi² = 1.58, df = 1 (P = 0.21); I² = 37%						
Test for overall effect: Z = 2.85 (P = 0.004)						
**1.3.3 Solid tumors**						
Heck et al 2020	265	4319	10302	195872	100.0%	1.18 [1.04, 1.34]
Linet et al 1996	11	570	43	2850	0.0%	1.28 [0.66, 2.51]
**Subtotal (95% CI)**		**4319**		**195872**	**100.0%**	**1.18 [1.04, 1.34]**
Total events	265		10302			
Heterogeneity: Not applicable						
Test for overall effect: Z = 2.54 (P = 0.01)						
**1.3.4 Skin tumors**						
Berg 1997	2	30	11	120	0.0%	0.71 [0.15, 3.38]
**Subtotal (95% CI)**		**0**		**0**		**Not estimable**
Total events	0		0			
Heterogeneity: Not applicable						
Test for overall effect: Not applicable						

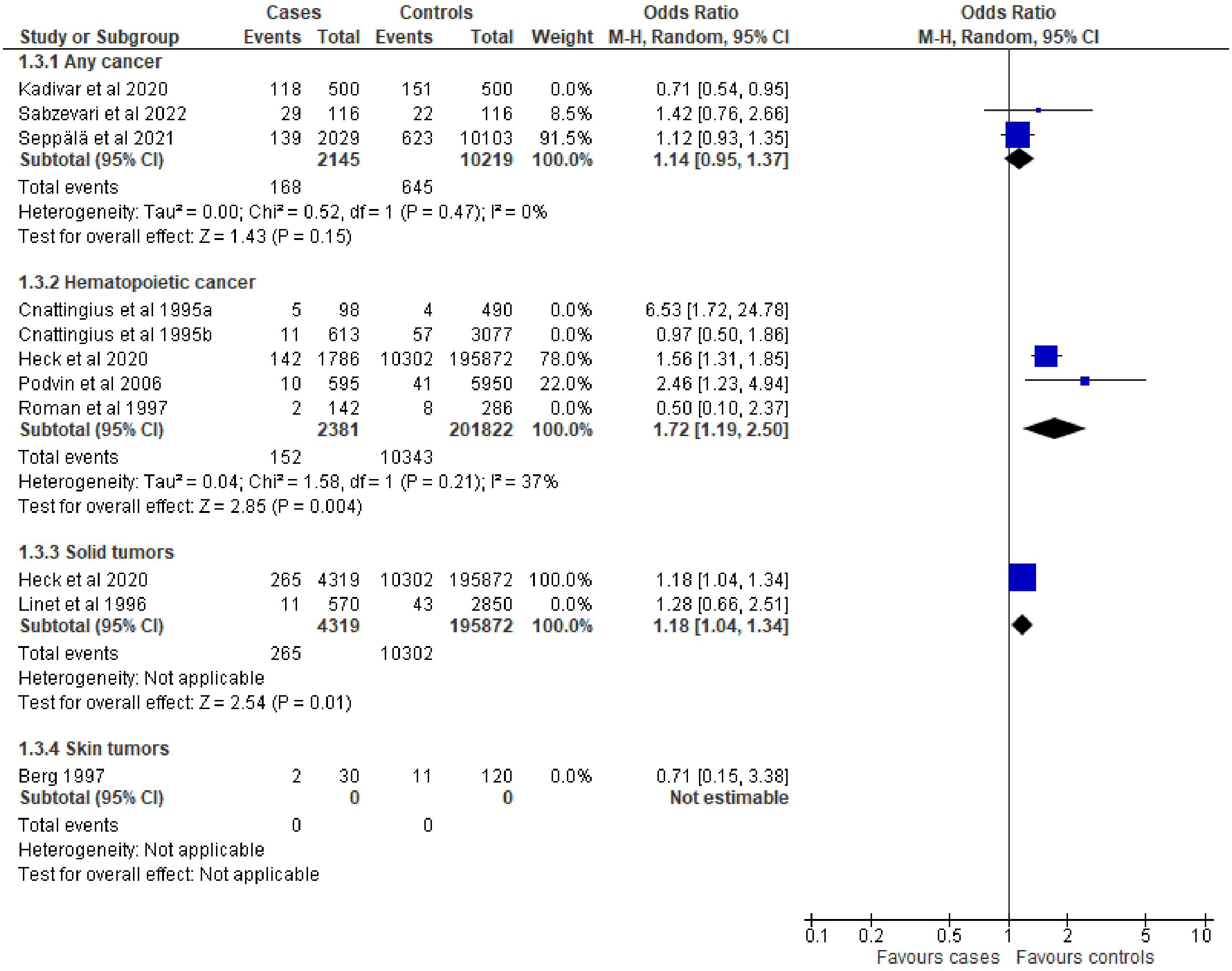

Figure S4 Causal pathways illustrated in directed acyclic graph. The graph presents possible confounders and modifiers for the causal pathway between phototherapy and cancer.

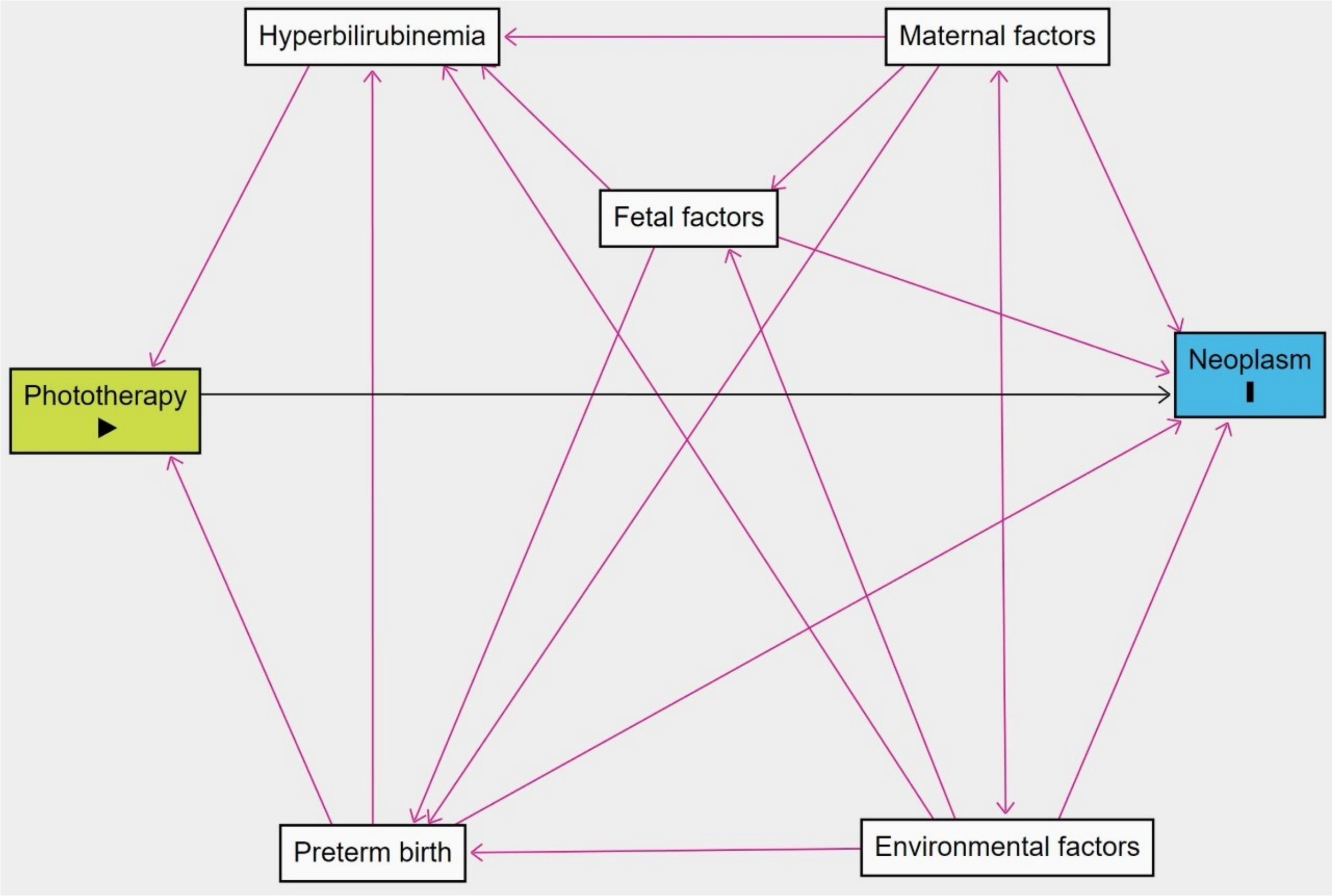

Supplement: Supplementary file 3 — Supplementary material [file 41390_2024_3191_MOESM3_ESM.pdf]
